# Kinetic and thermodynamic investigation of the removal of alizarin red dye using silica-supported nanoscale zero-valent iron particles

**DOI:** 10.1038/s41598-025-15233-z

**Published:** 2025-08-26

**Authors:** Ibrahim El-Hallag, Ahmad Al-Owais, El-Sayed El-Mossalamy

**Affiliations:** 1 Chemistry Department, Faculty of Science, Tanta University, Tanta, Egypt; 2 Chemistry Department, College of Science, King Saud University, Riyadh, Saudi Arabia; 3 Chemistry Department, Faculty of Science, Benha University, Benha, Egypt

**Keywords:** Alizarin dye, Adsorption, Nanocomposites, Langmuir model, Wastewater, Chemistry, Materials chemistry

## Abstract

Alizarin red (ARS) dye is a persistent and toxic pollutant in aquatic environments, posing a significant environmental threat. Among various treatment technologies, adsorption offers a practical and highly efficient method for dye removal. This study investigates the application of silica-supported nanoscale zero-valent iron (nZVI) particles as adsorbents for the removal of ARS dye, with a focus on evaluating the adsorption kinetics and thermodynamic behavior of the process. Two nanocomposites were synthesized using iron precursors with different counter ions: chloride (nZVI/Cl⁻) and nitrate (nZVI/NO₃⁻). The choice of counter ion influenced the physicochemical characteristics of the materials, thereby affecting their adsorption efficiency. The effects of key operational parameters, including solution pH, temperature, contact time, and adsorbent dosage, were systematically examined. Optimal adsorption was observed at pH 3, achieving removal efficiencies of 94.9% for nZVI/Cl⁻ and 85.0% for nZVI/NO₃⁻. Adsorption isotherm analysis revealed that the data fit well to the Langmuir model, indicating monolayer adsorption onto a homogeneous surface. Thermodynamic parameters confirmed that the adsorption process is spontaneous and endothermic in nature. These results underscore the potential of silica-supported nZVI nanocomposites as effective, sustainable, and environmentally friendly adsorbents for the remediation of dye-contaminated wastewater.

**Supplementary Information:**

The online version contains supplementary material available at 10.1038/s41598-025-15233-z.

## Introduction

Significant environmental concerns have been raised by the discovery that alizarin dye, a synthetic colourant widely used in the textile industry, is a persistent pollutant in aquatic systems^1^. Alizarin^2^ and other non-biodegradable substances make up a significant amount of the synthetic dyes that are thought to be released annually by the worldwide textile industry. Alizarin’s known mutagenic and carcinogenic qualities exacerbate the environmental dangers connected with it^3–5^. Thus, it is essential to look into effective and durable ways to remove it from watery environments. Adsorption has drawn a lot of interest among the current treatment techniques because of its ease of use, economic feasibility, and high removal efficacy for a variety of dye pollutants^6,7^. Notably, its ability to eliminate colours at low concentrations makes it especially appropriate for environmental restoration and industrial wastewater control^8–10^. Adsorption’s versatility, which results from adjustable process parameters and a huge range of adsorbents, increases its allure for large-scale applications^11^.

Alizarin Red dye is resistant to biodegradation and conventional wastewater treatment; therefore, it remains in water bodies^12,13^. HPLC, UV-Vis, and LC-MS are commonly used detection techniques. Toxically, it can induce oxidative stress, membrane damage, and metabolic disruption in aquatic creatures, with possible bioaccumulation hazards extending to higher trophic levels, including humans^14–18^. Alizarin red offers significant environmental and health problems since it has the capacity to affect organismal development, reproduction, and survival.The textile industry, particularly dyeing and finishing processes, accounts for approximately 20% of global industrial water pollution, with developing countries being especially vulnerable due to inadequate wastewater treatment infrastructure, resulting in the direct discharge of dye-laden effluents into water bodies^16,17^.

The use of nanostructured materials in dye adsorption has shown a lot of promise in recent years^19,20^. Nanoparticles can interact with dye molecules like alizarin red more efficiently due to their high surface-to-volume ratios, variable surface chemistry, and availability of reactive sites^21,22^. Even at low concentrations, these characteristics enable effective dye uptake^23^. They are supported by scalable synthesis pathways and functionalisation techniques that enhance performance and specificity^24,25^. Furthermore, their employment as environmentally benign adsorbents is supported by their minimal environmental impact and capacity for regeneration and reuse^26^. With the added advantages of operational convenience and the potential for water reuse after treatment, adsorption has been shown in numerous studies to be a dependable and effective colour removal technique^27–30^. Process sustainability is further improved by the fact that adsorbents can frequently be recovered and repurposed^31–33^. Gold nanoparticle-modified activated carbon, FeO₃/activated carbon hybrids, polypyrrole-coated magnetic nanoparticles, and biomass-derived sorbents such as mustard husk^34,35^ are just a few of the many adsorbent materials that have been investigated. Among nanomaterials for environmental cleanup, nanoscale zero-valent iron (nZVI) has garnered more attention due to its capacity to efficiently break down and immobilise dangerous substances^36^. In polluted matrices, its large specific surface area improves reaction kinetics, and its nanoscale size permits increased mobility and dispersion. The viability of nZVI for field-scale applications has increased due to recent developments in synthesis processes that have further reduced production costs. The kinetics and thermodynamic characteristics of alizarin dye removal from aqueous media utilising silica-supported nanocomposites of zero-valent iron (nZVI) are examined in this work. The nature and viability of the adsorption process and thermodynamic analyses, including evaluations of entropy change (ΔS), enthalpy change (ΔH), and Gibbs free energy (ΔG), were also carried out.

SEM imaging, BET surface area measurements, and pore size studies were used in this investigation to indicate the effect of silica supported nZVI on its enhancement of the physicochemical characteristics and adsorption behavior for alizarin removal. The silica support can increase the surface area available to target contaminants, decrease nZVI agglomeration, and improve particle dispersion^37^. Additionally, silica’s mesoporous structure facilitates improved mass transfer and dye diffusion, especially for relatively big organic molecules like alizarin red dye^38,39^.

## Materials and methods

All of the compounds used in this investigation were analytical grade and were utilised exactly as supplied, requiring no additional purification. Sigma-Aldrich was the source of the sodium borohydride (NaBH₄), anhydrous ferric chloride (FeCl₃), ferric nitrate [Fe(NO₃)₃·9 H₂O], and alizarin red dye. Every experimental technique used deionised water.

### Synthesis of nZVI nanoparticles

To prepare the salts of nanoscale zero valent iron ( nZVI/Cl^−^ and nZVI/NO^−^ _3_) from different anions such as chloride (Cl¯) and nitrate (NO_3_^–^), a 0.6 M solution of each salt was dissolved in a 4/1 (v/v) ethanol/water mixture (24 ml ethanol + 6 ml deionized water) and stirred well^40–43^. On the other hand, a 0.8 M sodium borohydride solution was made by dissolving 3.028 g NaBH4 in 100 ml deionized water; excess borohydride is required for better growth of iron nanoparticles. The borohydride solution is poured into a burette and added to each solution drop by drop (1 drop per 2 s) while vigorously stirring. Immediately after the first drop of sodium borohydride solution, black solid particles emerged, and the remaining sodium borohydride solution was added completely. After adding the entire borohydride solution, the mixture was stirred for another 30 min. The black iron nanoparticles were quickly filtered to get rid of nanoparticles using a 0.45 μm syringe filter device, and its remaining dye concentration was measured. To eliminate all of the water, the solid particles were rinsed three times with 25 mL portions of absolute ethanol. Because it avoids the quick oxidation of zero valent iron nanoparticles. After that, the precipitate was placed in an autoclave at 160 °C for 4 h. Finally, the produced nanoparticles were dried overnight at 60 °C. A thin layer of ethanol was applied to protect the nano iron particles from oxidation during storage.

The idealized stoichiometric equation for the reduction of Fe³⁺ is:

$${\mathbf{2F}}{{\mathbf{e}}^{{\mathbf{3}}+}}+{\text{ }}{\mathbf{3NaB}}{{\mathbf{H}}_{\mathbf{4}}}\,+\,{\mathbf{9}}{{\mathbf{H}}_{\mathbf{2}}}{\mathbf{O}} \to {\mathbf{2F}}{{\mathbf{e}}^{\mathbf{0}}} \downarrow +{\text{ }}{\mathbf{3NaB}}{{\mathbf{O}}_{\mathbf{2}}}+{\text{ }}{\mathbf{12}}{{\mathbf{H}}_{\mathbf{2}}} \uparrow$$

### Adsorption experiments

The synthesised silica-supported nZVI composites for adsorbing alizarin red S (ARS) dye were systematically investigated using a variety of parameters, such as dye concentration, pH, temperature, adsorbent dose, and contact time. In a horizontal shaker set to 410 rpm, 50 mL of ARS solution (starting concentration: 150 mg/L) was combined with a known mass of adsorbent (0.1 g).

### Preparation of Alizarin red S (ARS) solution

Stock solutions of Alizarin Red S (ARS) were prepared at a concentration of 500 mg/L using deionized water. Serial dilution was used to create working solutions with the appropriate concentrations for batch experiments. To avoid contamination, all glassware was rinsed with deionised water after being acid-washed. Depending on the desired value, 0.1 M NaOH or 0.1 M HCl was used to measure and alter the dye solutions’ starting pH. Because pH has a major impact on both adsorbate speciation and adsorbent surface charge, the pH was continuously measured using a calibrated pH meter before and after each experiment to assure stability. At certain intervals, samples were taken out to track decolorisation.

### Adsorption kinetics evaluation & sample intervals

For kinetic studies, a fixed dose of nZVI composite (0.1 g) was added to 100 mL of ARS solution at a fixed initial concentration (50 mg/L) and agitated in a temperature-controlled shaker at 410 rpm and a constant temperature of 25 °C. Samples were collected at predetermined time intervals (5, 10, 20, 30, 60, 90, 120, and 180 min).

### Adsorption thermodynamics evaluation

Thermodynamic investigations were conducted with the same beginning dye concentration, pH, contact time, and adsorbent dose but at different temperatures (308, 318, 333, 340, and 350 K). Each temperature’s equilibrium adsorption data were utilised to calculate thermodynamic parameters, including ΔH, ΔS, and ΔG, using the van’t Hoff equation. Therefore, using ideal contact times of 180 min for nZVI/Cl⁻ and 120 min for nZVI/NO₃⁻—as well as a fixed adsorbent dosage of 0.1 g—temperature effects were evaluated at 308, 318, 333, 340, and 350 K. The standard formula found in earlier research was used to determine the dye clearance efficiency^40–44^.

1 $$\:T=\:\:\:\frac{(C{\:}_{i}-C{\:}_{f})}{m}\:\:\:V\:$$

and percentage elimination

2 $$\:=\:\frac{C\:{\:}_{i}-C{\:}_{f}}{C\:{\:}_{i}}.100$$

where *C*_i_ represents the initial concentration of alizarin red S (ARS) dye in solution (mg·L⁻¹), *C*_f_​ is the dye concentration after the adsorption process (mg·L⁻¹), V is the volume of the dye solution (L), m is the mass of the adsorbent utilised (g), and T is the amount of dye adsorbed per unit mass of the nZVI adsorbent (mg·g⁻¹).

### Determination of residual dye concentration

UV-Vis spectrophotometry was used to assess the residual concentration of Alizarin Red S (ARS) in solution following adsorption. Following each adsorption run, the solutions were filtered through a 0.45 μm membrane filter device to remove suspended adsorbent particles. The clear filtrate was analysed using a Shimadzu UV-1800 UV-Vis spectrophotometer, taking absorbance data at 420 nm, the maximum absorption wavelength (λ_max) of ARS. The determination of the residual concentration of Alizarin Red S (ARS) in solution is performed via constructing a calibration curve for ARS solutions with concentrations ranging from 0 to 25 mg·L⁻¹, which showed excellent linearity (R² = 0.9992). The limits of detection (LOD) and quantitation (LOQ) were found to be 0.25 mg·L⁻¹ and 0.75 mg·L⁻¹, respectively. All spectrophotometric studies were carried out in triplicate, with blank samples serving as references to account for any background absorbance.

### SEM experiment

The surface structure of the investigated adsorbent was conducted via a field emission scanning electron microscopy (FE-SEM, QUANTAFEG 250).

### BET surface area experiment

The various surface parameters of the prepared nZVI were determined from N_2_ adsorption-desorption isotherms measured at -196 °C using Brunauer-Emmet-Teller (BET) method with a NOVA 2200 instrument (Quantachrome, USA). The samples were initially outgassed under vacuum at 300 °C for 1 h. BET surface areas (SBET) were calculated by the aid of the BET Eq. (9).

## Results and discussion

### Influence of pH on ARS dye adsorption

**Fig. 1:**
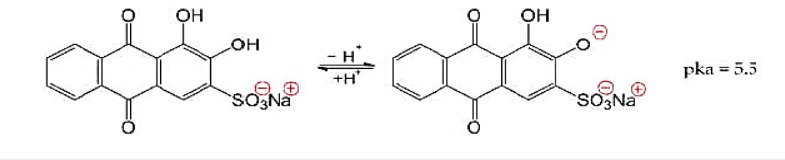
Acid–base equilibrium of Alizarin red S (ARS).

Electrostatic interactions between functional groups on the adsorbent surface and charged dye molecules dominate the adsorption behavior of ARS dye on nZVI composites^45^. Because both the adsorbent and dye can gain or lose charge depending on the pH of the solution, this parameter is essential in determining the degree of adsorption. The phenolic group in ARS dye dissociates at roughly 5.5 pKa, altering its charge distribution in solution (Fig. 1)^46,47^. A previous study has shown that the adsorption effectiveness of dyes is greatly controlled by the pH of the medium, which affects the dye’s ionisation state as well as the adsorbent’s surface charge^48^.

**Fig. 2:**
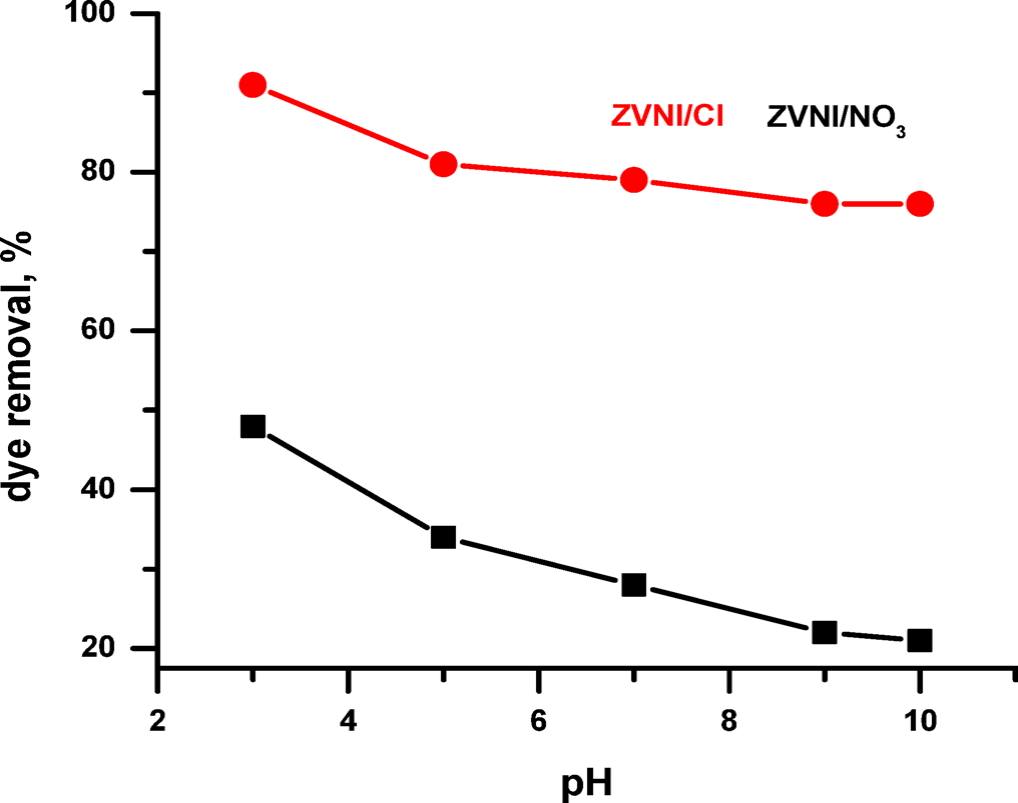
Effect of pH on the adsorption efficiency of ARS dye using nZVI composites as adsorbents (initial ARS concentration: 500 mg·L⁻¹).

Furthermore, it was found that the optimum adsorption pH changes according to the unique features of each adsorbent-adsorbate system^49^. In this investigation, the dye solution’s starting pH was varied from 3.0 to 10.0 to assess its effect on adsorption (Fig. 2). The findings revealed that the greatest adsorption occurred at pH 3.0, where the nZVI nanocomposites had the highest affinity for ARS. At this pH, the nZVI/Cl⁻ composite had a dye removal efficiency of 89.9%, while the nZVI/NO₃⁻ version had 45%. This pH-dependent trend is caused by variations in the surface charge of the nZVI materials as well as the ARS dye’s ionization state. Under acidic conditions, nZVI’s surface is likely positively charged, which promotes electrostatic attraction with anionic ARS molecules. As the pH rises, the adsorbent surface becomes less positively charged, even negatively charged, weakening interactions and reducing adsorption efficacy.

Accordingly, as explained before, the adsorption behavior of alizarin red S (ARS) on nZVI composites is pH dependent and may be explained by examining the dye’s ionization state, the adsorbent’s surface charge, and the nature of electrostatic interactions. ARS is an anionic dye with phenolic and sulfonic acid groups that deprotonate in aqueous solution as the pH changes. At low pH (pH < 5.5), ARS is mostly protonated or slightly deprotonated, while at higher pH, it is entirely deprotonated and negatively charged. Simultaneously, the surface of nZVI is positively charged in acidic circumstances due to protonation of its hydroxyl groups. This causes a significant electrostatic interaction between the positively charged nZVI surface and the negatively charged ARS molecules, facilitating more adsorption^50–52^. In contrast, the slightly oxidising character of nitrate ions in nZVI/NO₃⁻ leads to enhanced surface oxidation and Fe(III) oxide production, which reduces Fe⁰ availability and imparts a greater negative surface charge under alkaline circumstances, decreasing both ARS adsorption and dye removal efficacy^53–55^.

### Thermodynamic analysis

**Fig. 3:**
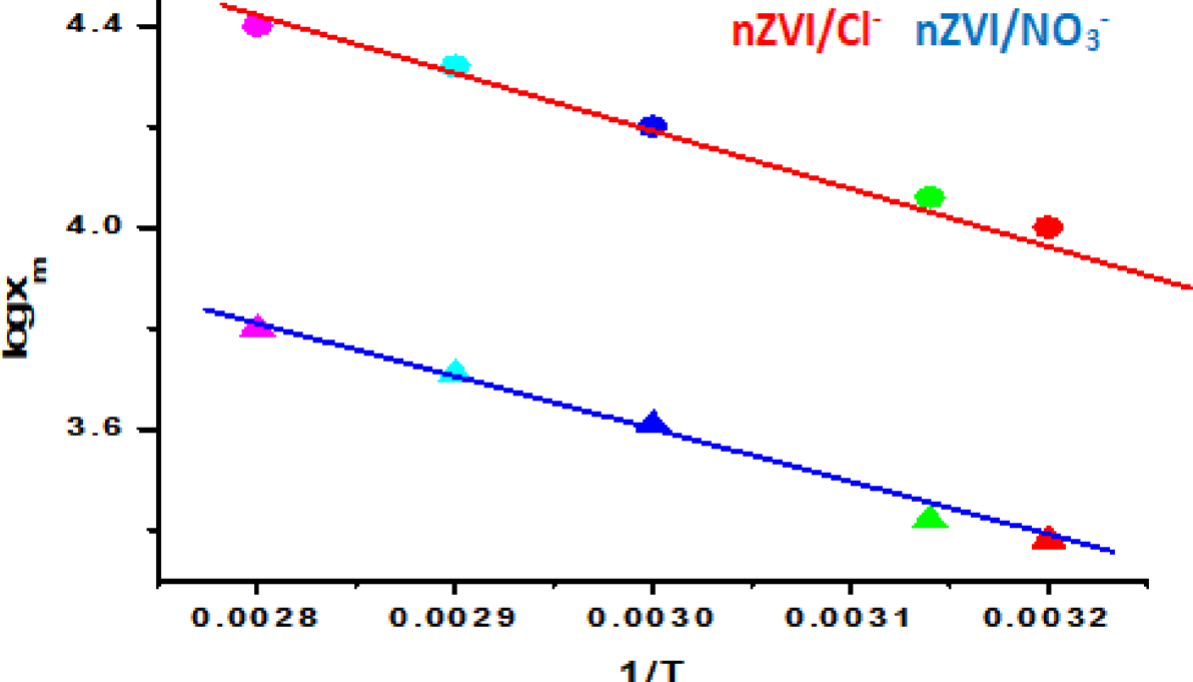
presents the relationship between log Xm and the reciprocal of temperature (1/T) for the adsorption of alizarin red dye onto silica-supported nZVI/Cl⁻ and nZVI/NO₃⁻ nanocomposites.

As mentioned before, the experiments with nZVI/Cl⁻ and nZVI/NO₃⁻ composites were carried out at different temperatures (308, 318, 333, 340, and 350 K) to study the thermal behavior of the ARS dye adsorption process. The data show that adsorption capacity increases with temperature, assuming that the process is endothermic. The computed positive enthalpy change (ΔH) supports this fact, indicating both adsorption and absorption mechanisms are involved. An increase in temperature increases the kinetic energy of ARS dye molecules, increasing diffusion and deeper penetration into the adsorbent’s pores. Furthermore, the enhanced interaction between the adsorbent and dye molecules at higher temperatures implies a stronger binding affinity, which leads to increased absorption. An earlier study employed classic thermodynamic Eqs. (3 & 4) to calculate thermodynamic parameters, including entropy change (ΔS), Gibbs free energy change (ΔG), and enthalpy change (ΔH)^56^. These factors add to our understanding of the adsorption process’s feasibility, spontaneity, and energetics.

3 $$\:\text{log}\:{x}_{m}=\frac{-\varDelta\:H}{2.303\:RT}+\:\frac{\varDelta\:S}{R}\:\:$$

4 $$\:\varDelta\:G=\:\varDelta\:H-T\varDelta\:S\:$$

where Xm is the largest amount of adsorbate (mg/g), *R* = 8.314 J/mol K (gas constant), and T, measured in Kelvin, is the absolute temperature. As illustrated in Fig. 3, ΔH is identified by the van Hoff plot’s slope (log(X_m_) vs. 1/T), and ΔS was identified by the intercept.

**Fig. 4:**
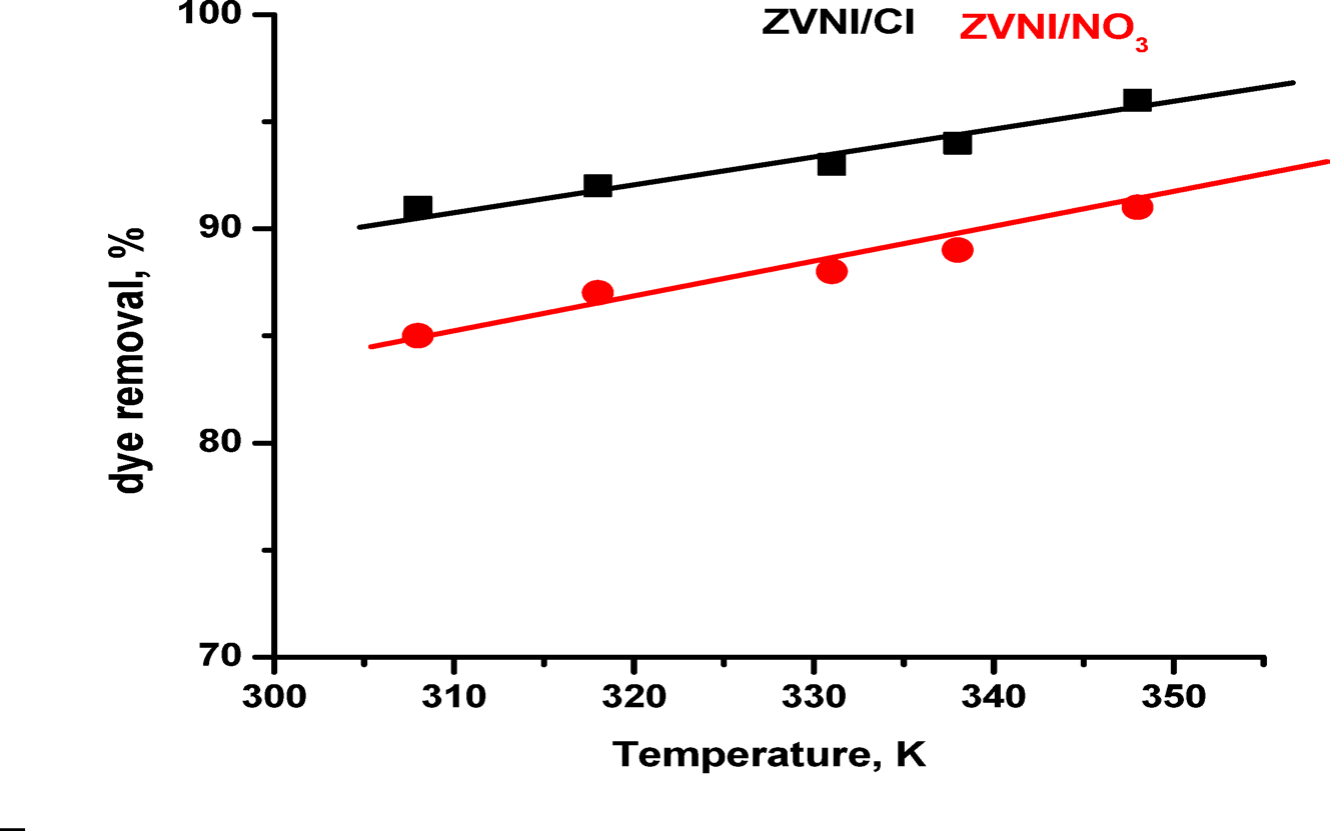
Influence of temperature on the removal efficiency of alizarin red dye using nZVI/Cl⁻ and nZVI/NO₃⁻ nanocomposite adsorbents.

The thermodynamic evaluation yielded computed enthalpy change (ΔH) and entropy change (ΔS) values of 24.26 kJ/mol and 69.94 kJ/(mol·K) for nZVI/Cl^−,^ while for nZVI/NO_3_^−^ it yielded 18.34 kJ/mol and 54.9 kJ/(mol.K). These data indicate that the ARS dye molecules have continuous surface mobility, implying the occurrence of both adsorption and absorption processes. The calculated negative values of Gibbs free energy change (ΔG) for nZVI/Cl^−^ and nZVI/NO_3_^−^ are − 3.418 kJ/mol and − 1.624 kJ/mol, respectively, confirming that the adsorption of ARS onto the surfaces of nZVI/Cl⁻ and nZVI/NO₃⁻ is spontaneous. The magnitude of ΔG decreased with increasing temperature, demonstrating a thermodynamically favorable adsorption process.Adsorption interactions can be characterized broadly based on enthalpy values: (a) physical adsorption for ΔH < 20 kJ/mol, (b) electrostatic interaction for ΔH ≤ 80 kJ/mol, and (c) chemisorption for ΔH ≤ 450 kJ/mol^56^. Positive ΔH values (20–80 kJ/mol) imply endothermic adsorption. This shows that the adsorption method involves interactions requiring energy input, such as overcoming surface hydration layers or enabling surface functional group rearrangement. The moderate ΔH value indicates physical adsorption through electrostatic and hydrogen bonding interactions rather than substantial chemisorption^57^. The positive ΔS indicates enhanced randomization at the solid-liquid interface, which is consistent with dye molecules being displaced from the aqueous phase onto the solid adsorbent surface^58–60^. Figure 4 shows the adsorption efficiency of ARS dye at varying temperatures using nZVI/Cl⁻ and nZVI/NO₃⁻ adsorbents. As shown, greater temperatures increase dye removal, reinforcing the endothermic nature of the adsorption process.

The larger ΔS value for nZVI/Cl⁻ suggests a more entropy-favourable interaction, possibly due to a less oxidised and more reactive surface compared to nZVI/NO₃⁻. Together, these findings show that ARS adsorption on nZVI-based composites is thermodynamically advantageous, endothermic, and influenced by entropy increases at the solid-liquid interface. The nZVI/Cl⁻ combination has a superior thermodynamic profile, resulting in higher experimental adsorption capacity and increased surface availability of Fe⁰. The following Table 1 summarizes the thermodynamic parameters obtained for Alizarin Red S (ARS) adsorption in this study and compares them with relevant values previously reported in the literature.

**Table 1:** Thermodynamic parameters enthalpy change (ΔH), entropy change (ΔS), and Gibbs free energy change (ΔG) for Alizarin red S adsorption on various adsorbents.

ΔH (kJ/mol)	ΔS (J/mol·K)	ΔG (kJ/mol)	References
+ 24.26	+ 69.94	− 3.418	This work (nZVI/Cl^−^)
+18.34	+54.9	− 1.624	This work (nZVI/NO_3_^−^)
21.41	0.076	− 1.800	[61]
+50.606	+ 174.679	− 1.677	[62]
22.785	64.142	− 1.410	[63]
-9.85	-29.43	− 1.072	[64]

As shown in Table 1, the thermodynamic parameters found in this study are comparable with trends described in references^61–63^ but differ from those provided in reference^64^.The positive values of ΔH and ΔS observed in our system indicate that the adsorption process is endothermic and accompanied by an increase in randomness at the solid–solution interface. In contrast, the negative ΔH and ΔS values reported in reference^64^ correspond to an exothermic process with decreased entropy. The consistently negative ΔG values confirm the spontaneous nature of the adsorption. These results collectively suggest that the adsorption of alizarin red onto the nZVI–SiO₂ composite is primarily energy-driven and that increased temperature enhances dye uptake by promoting thermodynamically favorable interactions.

### Adsorption isotherms

Adsorption isotherm models were used to analyze the equilibrium data to better understand the interaction between ARS dye molecules and the surface of the synthesized nanocomposite. The Langmuir and Freundlich isotherms, two commonly used models, were used to analyze the experimental results.

### Langmuir isotherm model

The Langmuir model is based on the assumption that adsorption occurs equally across a surface comprised of identical patches, resulting in a monolayer covering in which the adsorbed molecules do not interact^58^. Furthermore, this model assumes that a dye molecule cannot be adsorbed at a site after it has established itself there. Equation (5) describes the mathematical form of the Langmuir Eq. 

5 $$\:\frac{\:{C}_{e}}{\:{Q}_{e}}=\:\frac{1}{a}\:\:+\:\frac{b}{a}\:{C}_{e}\:$$

**Fig. 5:**
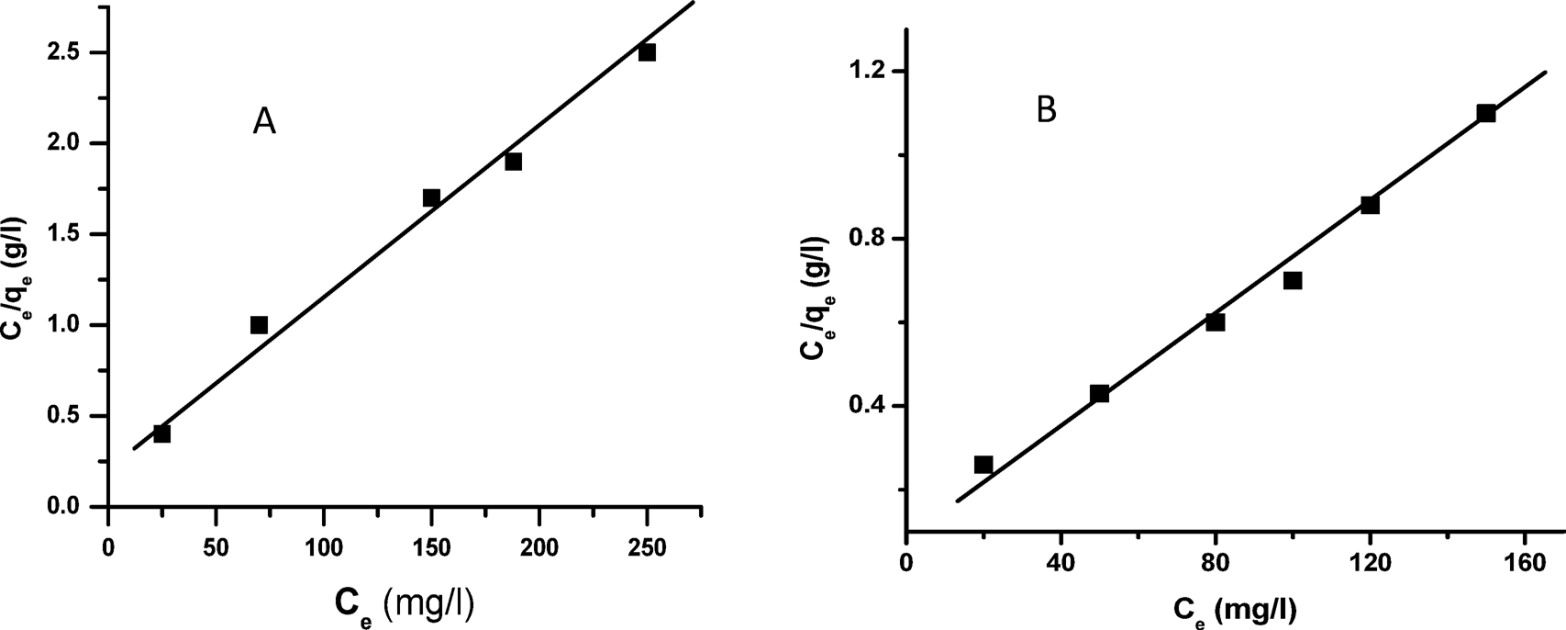
Langmuir isotherm for the adsorption of alizarin red dye on nZVI/Cl^-^ (**A**) and on nZVI/NO^-^_3_ (**B**).

In the Langmuir model, *C*_e_ reflects the dye’s equilibrium concentration in the liquid phase, whereas (*a*) and (*b*) are Langmuir constants related to adsorption energy. *Q*_e_ is the equilibrium adsorption capacity of alizarin red dye on nZVI/Cl⁻ and nZVI/NO₃⁻ composites. Figure 5 illustrates the connection between (*C*_e_/*Q*_e_) and (*C*_e_), which was used to fit the adsorption data to the Langmuir model for the nZVI/Cl⁻ and nZVI/NO₃⁻ composites. The results of monolayer adsorption onto a surface with identical sites and homogeneous adsorption energy reveal that the adsorption behavior is consistent with the Langmuir Eqs. 5^6, 58^.

As shown, the experimental data fit the Langmuir model well, with correlation coefficients (R²) more than 0.98 for both composites (Fig. 5). This good match implies that monolayer adsorption is dominant and that the ARS molecules likely occupy specific active spots on the nZVI surface without much lateral contact once adsorbed. The surface chemistry of newly synthesized nZVI composites, particularly those stabilized with Cl^−^ shows a consistent distribution of reactive Fe⁰ and hydroxylated iron oxide/hydroxide sites. These sites give consistent binding energy for negatively charged ARS anions, hence validating the Langmuir model’s assumption of homogeneous surface site distribution. Furthermore, no multilayer development or dye-dye aggregation was seen on the surface, consistent with the Langmuir framework’s non-interacting adsorbate assumption. This behavior is especially important in nZVI, where rapid surface passivation might inhibit the creation of multilayers or complicated surface clusters.

### Freundlich isotherm model

The Freundlich equation is given as follows:

6 $$\:\varvec{l}\varvec{n}\:{\varvec{q}}_{\varvec{e}}=\varvec{l}\varvec{n}\:{\varvec{K}}_{\varvec{f}}+\:\frac{1}{\varvec{n}}\:\varvec{l}\varvec{n}{\varvec{C}}_{\varvec{e}}\:$$

$${\varvec{K}}_{\varvec{f}}=\:\frac{\:{\varvec{q}}_{\varvec{m}}}{\:{\varvec{C}}_{\varvec{o}}^{1/\varvec{n}}}$$

**Fig. 6:**
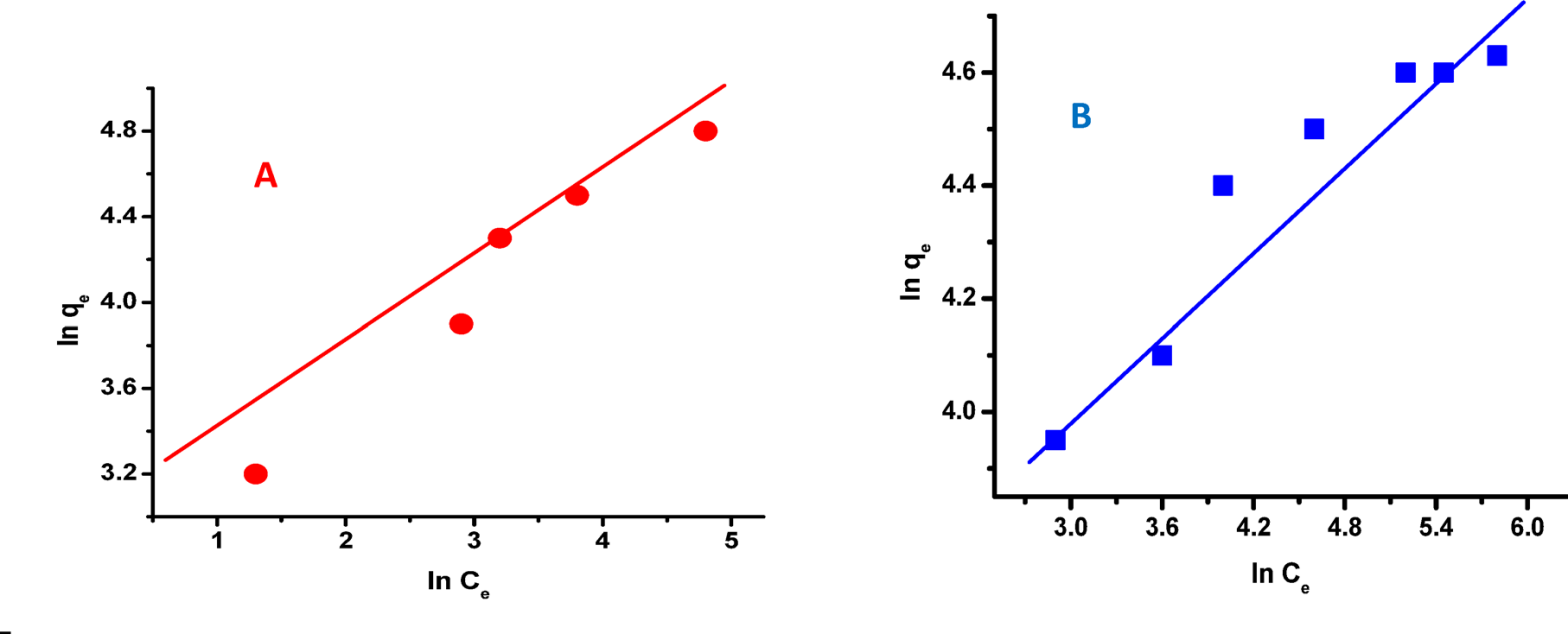
Freundlich isotherm for the adsorption of alizarin red dye onto nZVI/Cl⁻ (**A**) and nZVI/NO₃⁻ (**B**).

The constants *K*_f_ and n in the Freundlich model indicate the adsorption capacity and intensity, respectively. This model assumes a heterogeneous surface with variable energy adsorption sites and does not represent monolayer adsorption. Figure 6 shows the relationship between (ln *q*_e_) and (ln *C*_e_), which was used to fit alizarin red dye adsorption data to the Freundlich equation. The results show that the adsorption behavior does not conform to the Freundlich model.

The Freundlich isotherm model is an empirical equation that assumes adsorption happens on a heterogeneous surface with sites of different energies and that the adsorption process can involve multilayer development. This model demonstrated limited effectiveness in accurately projecting the adsorption equilibrium. This limitation may arise from the nature of the adsorbent surface, which might not exhibit significant heterogeneity, or from the predominance of monolayer adsorption rather than multilayer processes. However, as shown in Fig. 6, our experimental results clearly illustrate that the Freundlich model gives a poor match to the adsorption equilibrium data, as evidenced by low correlation coefficients and a lack of agreement between experimental and projected values. This shows that the analyzed adsorption systems do not meet the Freundlich model’s assumptions of energy heterogeneity or multilayer behavior. Interestingly, despite the nanocomposite material’s physically heterogeneous surface, the adsorption results suited the Langmuir isotherm model substantially better than the Freundlich model. This finding shows that functional adsorption behavior may be regulated by a more homogeneous group of active sites than surface morphology would indicate. One possible reason is that the surface functionalization or synthesis process resulted in a majority of certain, energetically comparable adsorption sites that dominate overall behavior^65,66^. Furthermore, adsorbate molecules may preferentially interact with accessible exterior sites due to steric effects or limited diffusion into microporous regions, substantially simplifying surface heterogeneity in terms of adsorption^67,68^. Additionally, the experimental conditions, particularly the concentration range used, likely emphasized monolayer coverage on high-affinity sites, which aligns with the assumptions of the Langmuir model^69,70^. This apparent contradiction between structural heterogeneity and Langmuir-type behavior highlights the importance of distinguishing between geometric and energetic heterogeneity in adsorption modeling.

**Adsorption Kinetics** Kinetic models were used to analyze the adsorption of alizarin red dye on nZVI/Cl⁻ and nZVI/NO₃⁻, based on experimental data. Kinetics describes the rate at which reactant or product concentrations change over time. The data were analyzed using the Eqs. (7) and (8) concerning the pseudo-first-order and pseudo-second-order kinetic models, respectively. Via 0.3 g of each nZVI/Cl⁻ and nZVI/NO₃⁻ adsorbents, dye adsorption reached equilibrium in around 80 minutes^59^. Table 2 compares the kinetic rate constants k_1_ and k_2_ of alizarin dye in our study to those reported in literature and adsorbed on nZVI and other adsorbents. As previously stated, our kinetic rate constant values are almost identical to those published in the literature for comparable systems, with minor variations likely related to differences in catalyst structure, reaction conditions, and mass transfer. The consistency supports comparable rate-determining steps, such as surface adsorption, intraparticle diffusion, or chemical transformation.

**Table 2:** Compare the values of kinetic rate constants of Alizarin dye in this work with those reported in literature and adsorbed at nZVI and other adsorbents.

Pseudo-first-order kinetic model		Pseudo-second-order kinetic model		References
K_1_ (min ^− 1^)	R^2^	K_2_ (g/mg.min)	R^2^	
0.015	0.816	0.398	0.997	This work (nZVI/Cl^−^)
0.011	0.802	0.325	0.975	This work (nZVI/NO_3_^−^
0.012	0.837	0.394	0.998	[71]
0.025	0.969	0.563	0.998	[72]

(pseudo-first-order model)

7 $$\:\text{\:\:ln}\left(qe-{q}_{t}\right)=\text{ln}qe-{k}_{1}\:t\:\:\:$$

(pseudo-second-order model)

8 $$\:\frac{1}{{q}_{t\:}}=\frac{1}{\:{k}_{2}\:{q}_{e}}\:\:+\frac{t}{{q}_{e\:\:}}\:$$

where *q*_e_ is the amount of red alizarin adsorbed in equilibrium (mg/g), *q*_t_ is the amount of red alizarin adsorbed at different periods *t* (mg/g), and *k*_1_ and *k*_2_ are the kinetic rate constant values. The pseudo-secondary order pattern accurately characterizes the cinematic data (R^2^ = 0.997) (Fig. 7).

**Fig. 7:**
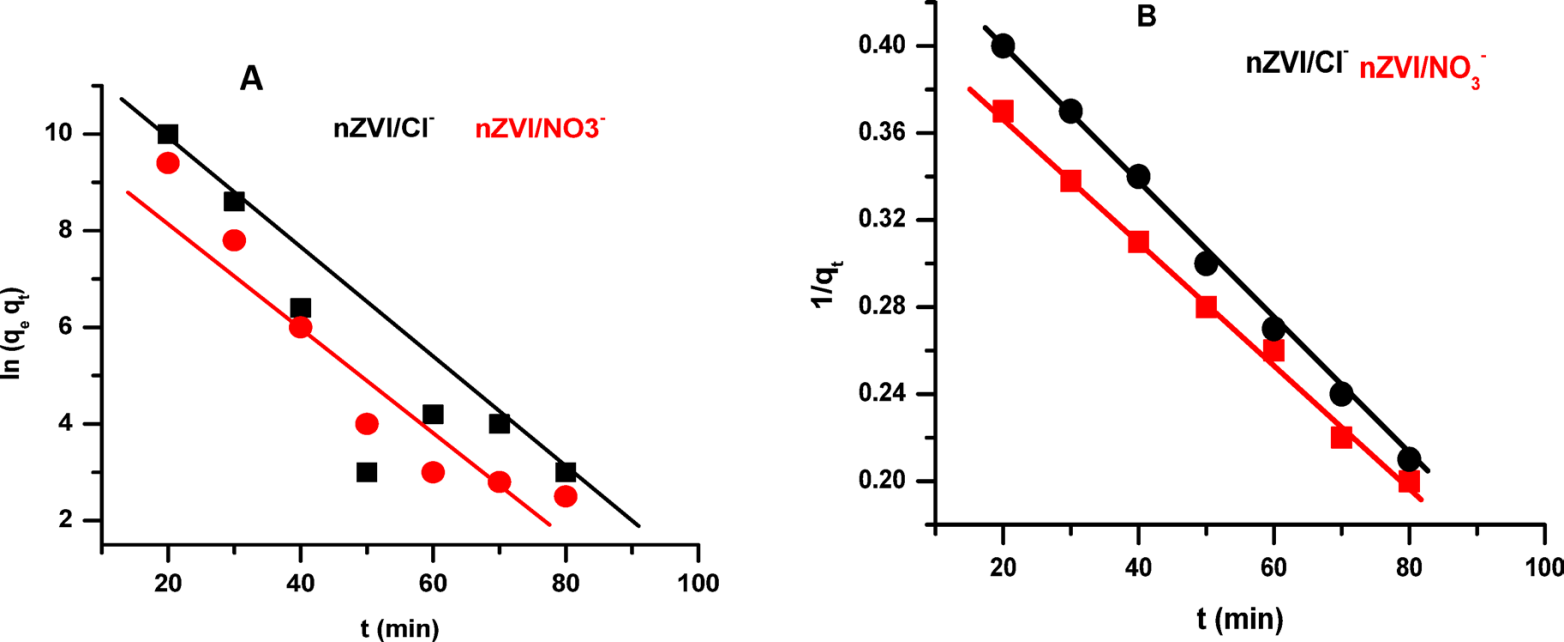
Dye adsorption kinetic curves, pseudo first order (**A**), pseudo second order (**B**).

The adsorption kinetics of ARS onto nZVI/Cl⁻ and nZVI/NO₃⁻ were better described by the pseudo-second-order (PSO) model (k₂ = 0.398 & 0.325 g·mg⁻¹·min⁻¹, R² > 0.99) for both nZVI/Cl⁻ and nZVI/NO₃⁻, respectively, indicating the rate-limiting step involves valence forces through electron sharing or exchange, which facilitate dye binding via electrostatic interactions, hydrogen bonding, and complexation mechanisms. In contrast, the pseudo-first-order model showed a lower rate constant (*k*₁ = 0.015 & 0.011 min⁻¹) for nZVI/Cl⁻ and nZVI/NO₃⁻, respectively, and a poor fit for both composites. The higher *k*₂ value and short equilibrium time (~ 80 min with 0.3 g adsorbent) confirm the strong interaction between ARS and the active sites of the nanocomposites, underscoring their potential for efficient dye removal in water treatment.

### Effect of adsorbent dose

**Fig. 8:**
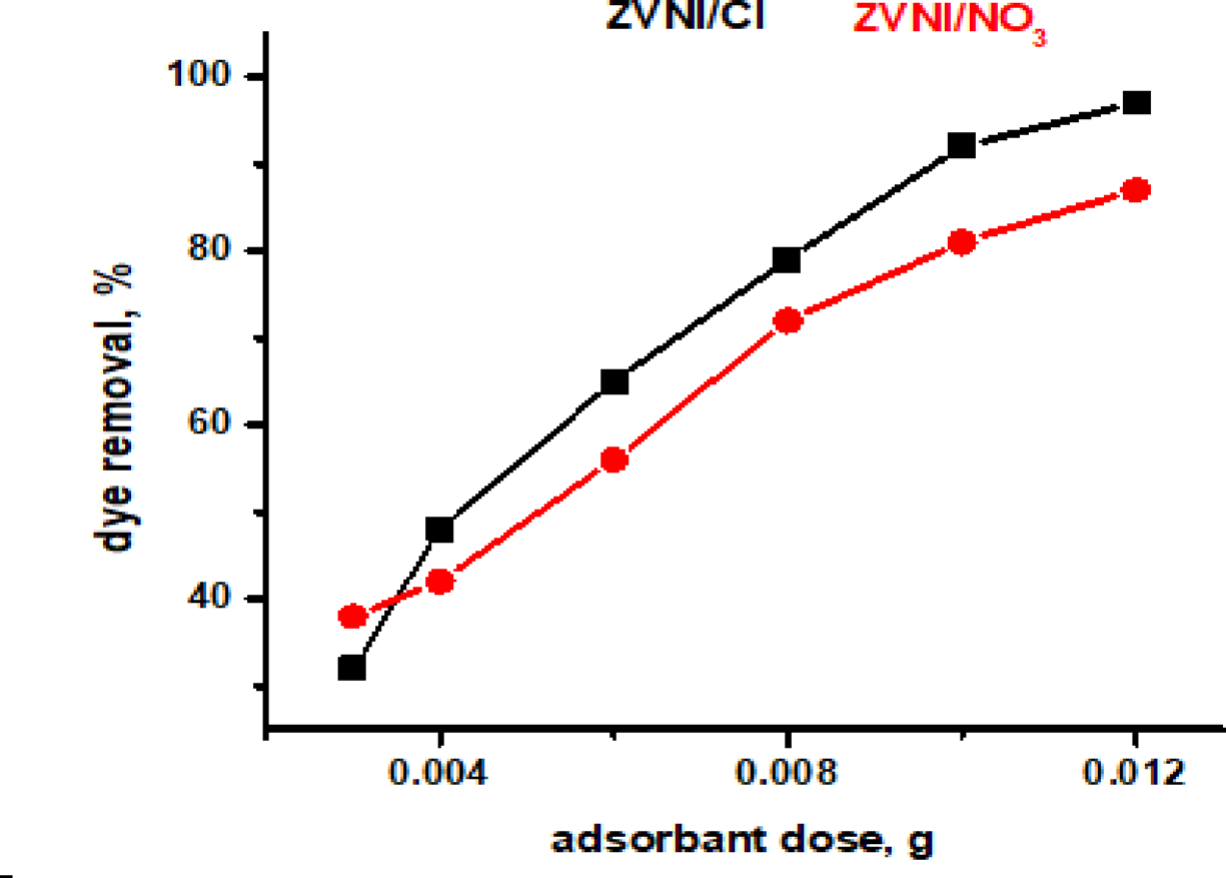
Effect of adsorbent dose on the alizarin red dye removal efficiency using nZVI/Cl^−^ and nZVI/NO^−^ _3_ adsorbents.

The adsorbent dosage is critical in determining adsorption capability for a particular adsorbate concentration. To study this, doses ranging from 0.002 to 0.012 g of adsorbent were tested to see how they affected alizarin red dye removal. The studies involved adding the adsorbent to 50 mL of alizarin red dye solutions at an initial concentration of 150 mg/L for both nZVI/Cl⁻ and nZVI/NO₃⁻. After the systems reached equilibrium, the solutions were centrifuged, and the dye concentrations were determined. Figure 8 shows that increasing the adsorbent dose improves dye removal efficiency, most likely because the adsorbent surface has more active sites. The dye removal efficiency was highest at 96.786% for nZVI/Cl⁻ and 87.206% for nZVI/NO₃⁻.

From Fig. 8, it was noticed that increasing adsorbent dosage initially resulted in a significant improvement in ARS dye removal efficiency due to increased availability of active sites. However, beyond an appropriate dosage, efficiency plateaued, indicating a saturation effect in which the majority of dye molecules had already been adsorbed and extra active sites remained underutilized. At higher dosages, particle agglomeration is likely to develop, limiting effective surface area and preventing access to active sites, diminishing adsorption capacity per unit mass. This behavior has important implications for determining the optimal dosage in large-scale applications. Overdosing not only leads to inefficient use of materials but may also hinder performance due to particle aggregation. Therefore, optimizing the dosage is essential to achieving a balance between maximum removal efficiency, effective utilization of active sites, and minimal agglomeration, ensuring the practicality and cost-effectiveness of the adsorption process.

While the primary goal of this investigation was to assess the removal effectiveness of alizarin red dye using freshly manufactured nZVI composites, we recognize that the long-term durability of such materials under varying environmental conditions is an important consideration for real-world applications. pH, temperature, oxidative exposure, and particle agglomeration all have a substantial impact on nZVI’s reactivity and overall performance. Previously published research work has demonstrated that nZVI particles are susceptible to surface oxidation and aggregation over time, particularly in aqueous systems with high oxygen concentration or neutral to alkaline pH levels, which can reduce their reactivity and mobility^73,74^. Elevated temperatures may hasten these degrading processes. Although long-term ageing tests were not included in the current study, future research should evaluate the structural and functional stability of nZVI composites over longer time periods, including under various environmental stresses. Such investigations are required to demonstrate that the material performs consistently, is storable, and can be deployed safely in environmental remediation settings.

Further, temperature variation plays a critical role in determining the adsorption capacity of nZVI composites. Our experimental results confirm that the adsorption of ARS onto both nZVI/Cl⁻ and nZVI/NO₃⁻ is endothermic in nature, with increased temperatures leading to higher adsorption capacities. This is attributed to enhanced molecular mobility and diffusion rates at elevated temperatures, which improve the transport of dye molecules to active surface sites. Additionally, higher temperatures may activate previously inaccessible or lower-energy adsorption sites through surface charge redistribution or structural changes. These effects collectively strengthen the adsorbate–adsorbent interaction. However, excessively high temperatures may result in increased desorption or compromise the structural integrity of the adsorbent. Therefore, identifying an optimal temperature range is essential for maximizing performance while ensuring long-term material stability and process efficiency.

### Determination of the residual dye concentration

The residual concentration of Alizarin Red S (ARS) in solution following adsorption was determined using UV-Vis spectrophotometry via the calibration method. This method is performed via constructing a calibration curve for ARS solutions with concentrations ranging from 0 to 25 mg·L⁻¹, which showed excellent linearity (R² = 0.9992). The limits of detection (LOD) and quantitation (LOQ) were found to be 0.25 mg·L⁻¹ and 0.75 mg·L⁻¹, respectively. All spectrophotometric studies were carried out in triplicate, with blank samples serving as references to account for any background absorbance.

### SEM experiment

**Fig. 9:**
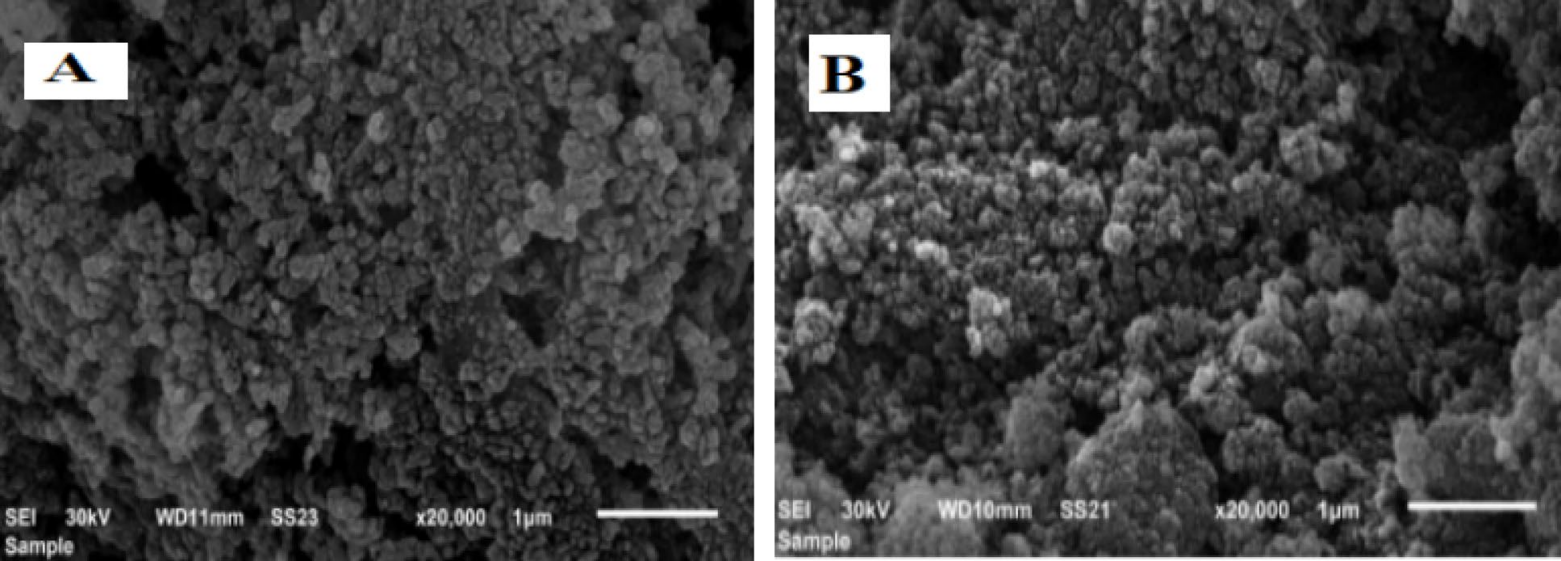
SEM images showing the morphology of the fabricated nZVI/Cl^−^ (**A**) and nZVI/NO_3_^−^ (**B**).

Figure 9 depicts the SEM images of the synthesised nZVI particles, which exhibit the surface morphology of the nZVI under consideration. The image shows that nZVI/Cl^−^ and nZVI/NO_3_^−^ particles have a core-shell configuration with particle sizes ranging from 20 to 70 nm. As depicted, the particles formed a chainlike, aggregated structure as a result of magnetic interactions and a natural desire to remain in a more thermodynamically stable condition. Iron nanoparticle aggregation is thought to be caused by magnetic dipole-dipole interactions and the individual particles’ large surface areas.

### BET measurements

The following equation can be used to calculate the specific surface area (SSA) of a spherical particle with a diameter (d):

9 $${\text{SSA }} = \, \left( {{\text{Surface Area}}/{\text{Mass}}} \right) = \frac{{{{(\pi d) }}^{2} }}{{{\raise0.7ex\hbox{${{{\rho \pi }}}$} \!\mathord{\left/ {\vphantom {{{{\rho \pi }}} {6}}}\right.\kern-0pt}\!\lower0.7ex\hbox{${6}$}}{\text{d }}^{3} }}= 6\pi /\rho d$$

where (*ρ*) represents the solid particle’s iron density (7.87 g/cm3). The surface area analyzer’s BET measurements produced SSA values of 13.62 and 9.76 m2/kg^2^ for nZVI/Cl^−^ and nZVI/NO_3_^−^, respectively. Furthermore, the synthesized nZVI/Cl^−^ and nZVI/NO_3_^−^ showed a BJH adsorption average pore diameter of 7.06 nm, which was consistent with the results of prior studies. In comparison to conventional iron powder, the synthesized nZVI had a considerably smaller diameter and a larger BET surface area. These modifications increased the nZVI activity, making it appealing for pollutant removal.

## Conclusion

This study demonstrates that pH, temperature, and adsorbent dosage significantly influence the adsorption of Alizarin Red S (ARS) onto silica-supported nZVI composites, with nZVI/Cl⁻ consistently outperforming nZVI/NO₃⁻, especially under acidic conditions (up to 89.9% removal). The process is pH-sensitive and thermodynamically endothermic and spontaneous, driven by electrostatic interactions and improved molecular mobility at higher temperatures. Adsorption followed the Langmuir isotherm (monolayer adsorption) and pseudo-second-order kinetics. While increased adsorbent dosage enhanced removal, excessive amounts led to aggregation and reduced efficiency. These findings highlight the potential of nZVI/Cl⁻ composites for anionic dye removal in wastewater treatment, stressing the importance of optimising operating conditions and investigating long-term stability and regeneration for real-world application. UV-Vis spectrophotometry was used to assess the residual concentration of Alizarin Red S (ARS) in solution after adsorption. The SEM image reveals that nZVI/Cl^−^ and nZVI/NO3^−^ particles have a core-shell configuration and diameters ranging from 20 to 70 nm. BET studies revealed that the synthesized nZVI had a significantly lower diameter and a greater BET surface area, indicating improved activity and effectiveness of the nZVI composite in eradicating alizarin Red S (ARS).

## Supplementary Information

Below is the link to the electronic supplementary material.

Supplementary Material 1

## Data Availability

The published article includes all the data presented in this study.
