# Supplementary material for: Kinetic and thermodynamic investigation of the removal of alizarin red dye using silica-supported nanoscale zero-valent iron particles
Source: Sci Rep. 2025 Aug 26;15:31461. doi: 10.1038/s41598-025-15233-z (PMC12381275; doi:10.1038/s41598-025-15233-z)
Supplement: Supplementary file 1 — Supplementary Material 1 [file 41598_2025_15233_MOESM1_ESM.pdf]

Kinetic and thermodynamic investigation of the removal of alizarin red dye adsorbed on nZVI

Ibrahim El-Hallag[*1], Ahmed Al-Owais[2], El-Sayed El-Mossalamy[*3]
[1]Chemistry Department, faculty of Science, Tanta University, Tanta, Egypt
[2]Chemistry Department, College of Science, King Saud University, Riyadh
[3]Chemistry Department, faculty of Science, Benha University, Benha, Egypt
*i.elhallag@yahoo.com
* alsayed.almosallamy@fsc.bu.edu.eg

Alizarin red dye is a toxic and long-lasting pollutant in water bodies, making it a serious environmental concern. Among the available treatment methods, adsorption offers a practical and efficient solution for its removal. This work explores the use of silica-supported nanoscale zero-valent iron (nZVI) particles as adsorbents to eliminate alizarin dye and the determination of the thermodynamic & kinetic parameters of adsorption and removal of alizarin dye. Two types of nanocomposites were made using iron salts with different counter ions—nitrate ([ZVNI/NO_3^-]) and chloride ([ZVNI/Cl^-]). These variations influenced the materials' performance, with the combined properties of the components enhancing their adsorption efficiency. Several factors affecting dye removal, such as pH, temperature, contact times, and adsorbent quantity, were thoroughly analyzed. The best performance was observed at pH 3, with removal rates reaching 94.9% for [ZVNI/Cl^-] and 85% for [ZVNI/NO_3^-]. It was concluded that adsorption followed the Langmuir model, indicating single-layer coverage on the surface. Thermodynamic results showed that the process is favourable and requires heat input, confirming it as endothermic. These outcomes demonstrate that silica-coated nZVI materials are effective and eco-friendly options for treating dye-contaminated wastewater.

Keywords: Alizarin dye; Adsorption; Nanocomposites; Langmuir model; Wastewater

Introduction

Alizarin dye, a synthetic colourant extensively utilized in the textile industry, has emerged as a persistent pollutant in aquatic systems, raising significant environmental concerns [1]. The global textile sector is estimated to discharge approximately 300,000 tonnes of synthetic dyes annually, a substantial portion of which comprises non-biodegradable compounds such as alizarin [2]. The environmental risks associated with alizarin are further intensified by its documented mutagenic and carcinogenic properties [3-5]. Therefore, investigating efficient and long-lasting methods for its removal from aqueous settings is imperative. Among the available treatment methods, adsorption has received considerable attention due to its operational simplicity, economic viability, and high removal efficiency for a broad range of dye pollutants [6, 7]. Notably, its capacity to remove dyes at low concentrations makes it particularly suitable for industrial effluent management and environmental restoration [8–10]. The adaptability of adsorption—stemming from tunable process conditions and the wide variety of available adsorbents—further enhances its appeal for large-scale applications [11].

In recent years, the application of nanostructured materials in dye adsorption has shown considerable promise [12, 13]. Because of their high surface-to-volume ratios, adjustable surface chemistry, and abundance of reactive sites, nanoparticles can interact with dye molecules like alizarin red more effectively [14, 15]. These properties facilitate efficient dye uptake, even at low concentrations [16], and are complemented by scalable synthesis routes and function-alization methods that improve specificity and performance [17, 18]. Moreover, their potential for regeneration and reuse, combined with low environmental impact, supports their use as environmentally benign adsorbents [19].

Numerous studies have demonstrated adsorption as a reliable and efficient strategy for dye removal, with the added benefits of operational convenience and the possibility of water reuse following treatment [20–23]. In many cases, adsorbents can be recovered and recycled, further improving process sustainability [24–26]. A variety of adsorbent materials have been explored, including gold nanoparticle–modified activated carbon, Fe_2O_3/activated carbon hybrids, polypyrrole-coated magnetic nanoparticles, and biomass-derived sorbents such as mustard husk [27, 28].

Because of its ability to effectively degrade and immobilise harmful chemicals, nanoscale zero-valent iron (nZVI) has attracted increased attention among nanomaterials for environmental remediation [29]. Its high specific surface area enhances reaction kinetics, while the nanoscale size allows for greater mobility and dispersion in contaminated matrices. Recent advancements in synthesis techniques have further lowered production costs, expanding the feasibility of nZVI for field-scale applications.

This study investigates the kinetics and thermodynamic parameters of the removal of alizarin dye from aqueous media using silica-supported zero-valent iron nanocomposites (ZVNI). The influence of key parameters—such as pH, temperature, contact time, adsorbent dosage, and adsorption kinetics—was systematically evaluated. Thermodynamic analyses, including

assessments of entropy change (ΔS), enthalpy change (ΔH), and Gibbs free energy (ΔG), were also conducted to elucidate the nature and feasibility of the adsorption process.

Materials and Methods

All chemicals used in this study were of analytical grade and used as received without further purification. Sodium borohydride ($NaBH_4$), anhydrous ferric chloride ($FeCl_3$), ferric nitrate [$Fe(NO_3)_3 \cdot 9H_2O$], and alizarin red dye were procured from Sigma-Aldrich. Deionised water was used in all experimental procedures.

Synthesis of nZVI Nanoparticles

The preparation of nanoscale zero-valent iron (nZVI) was carried out as established in literature [3]. The synthesis follows the general reduction reaction:

$$\mathbf{2FeX_n + 6NaBH_4 + 18H_2O \rightarrow 2Fe^0 + 6NaX + 6B(OH)_3 + 21H_2} \qquad (1)$$

Two variations of nZVI were synthesized using different anions—chloride ($NZVI/Cl^-$) and nitrate ($NZVI/NO_3^-$). For each, a 0.6 M solution of the iron precursor was prepared in a 4:1 ethanol-to-deionised water mixture (24 mL ethanol and 6 mL water) under continuous stirring. Separately, 3.028 g of $NaBH_4$ was dissolved in 100 mL of deionised water to form a 0.8 M reducing agent solution, ensuring an excess amount to promote complete reduction and enhance nanoparticle formation [30].

The $NaBH_4$ solution was added dropwise (approximately one drop every two seconds) into the iron salt solution using a burette under vigorous stirring. The formation of black precipitates marked the initiation of nanoparticle synthesis. After completing the dropwise addition, stirring was continued for an additional 30 minutes to ensure uniform particle formation. The resulting black suspension, containing nZVI particles, was separated via vacuum filtration.

To remove residual moisture and prevent oxidation, the particles were washed three times with 25 mL of absolute ethanol. This washing step is critical in maintaining the integrity of the zero-valent state of the iron nanoparticles. The filtered product was then autoclaved at 160°C for four hours, followed by overnight drying at 60°C. The final dried nanoparticles were stored under a thin ethanol layer to minimize oxidation during storage.

Adsorption Experiments

Using a number of variables, including dye concentration, pH, temperature, adsorbent dose, and contact time, the synthesised silica-supported nZVI composites' ability to adsorb alizarin red S (ARS) dye was methodically examined. A known mass of adsorbent (0.0012 g) was mixed with 50 mL of ARS solution (initial concentration: 150 mg/L) in a horizontal shaker set at 410 rpm. The pH was left unadjusted unless stated otherwise. Contact times were varied from 15 to 260 minutes. Samples were withdrawn at predetermined intervals to monitor decolorization.

Temperature effects were assessed at 308, 318, 333, 340, and 350 K using optimal contact durations—180 minutes for nZVI/Cl^- and 120 minutes for nZVI/NO_3^-—and a fixed adsorbent dosage of 0.012 g. Post-treatment, the mixtures were filtered and centrifuged to separate the adsorbent particles from the solution. The efficiency of dye removal was calculated using the standard formula as reported in previous literature [30].

$$T = \frac{(C_i - C_f)}{m} V \quad (1)$$

and percentage elimination

$$= \frac{C_i - C_f}{C_i} . 100 \quad (2)$$

where C_i represents the initial concentration of alizarin red S (ARS) dye in solution (mg·L⁻¹), C_f is the dye concentration after the adsorption process (mg·L⁻¹), V is the volume of the dye solution (L), m is the mass of the adsorbent utilised (g), and T is the amount of dye adsorbed per unit mass of the ZVNI adsorbent (mg·g⁻¹).

Results and Discussion

Influence of pH on ARS Dye Adsorption

The adsorption behavior of ARS dye on nZVI composites is largely governed by electrostatic forces among the functional groups on the adsorbent surface and the charged dye molecules [31]. Since both the adsorbent and the dye can gain or lose charge depending on the solution's pH, this parameter plays a critical role in determining the extent of adsorption.

ARS dye contains a phenolic group that dissociates with a pKa of approximately 5.5, influencing its charge distribution in solution (Scheme 1) [32, 33]. Previous research has highlighted that the adsorption efficiency of dyes is strongly influenced by the pH of the medium, because it influences the dye's ionisation state as well as the adsorbent's surface charge [34]. Moreover, the optimal pH for adsorption varies depending on the specific properties of each adsorbent–adsorbate system [35]. In this study, the initial pH of the dye solution was adjusted across a range from pH 3.0 to 9.0 to evaluate its effect on adsorption (Figure 1). The results demonstrated that

maximum adsorption occurred at pH 3.0, where the nZVI nanocomposites exhibited the highest affinity toward ARS. Specifically, dye removal efficiencies at this pH were found to be 89.9% for the [ZVNI/Cl^-] composite and 45% for the [ZVNI/NO_3^-] variant.

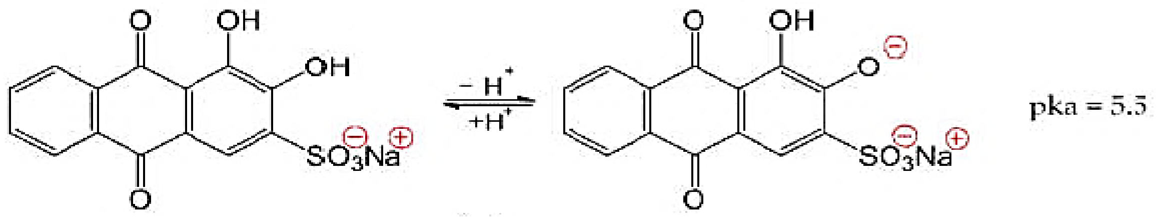

Scheme 1 Acid–base equilibria of (**a**) Alizarin red S (ARS)

This pH-dependent trend can be attributed to alterations in both the surface charge of the nZVI materials and the ionization state of the ARS dye. Under acidic conditions, the surface of nZVI likely carries a positive charge, which enhances electrostatic attraction with the anionic ARS molecules. As the pH increases, the adsorbent surface becomes less positively charged or even negatively charged, weakening the interactions and thereby reducing the adsorption efficiency.

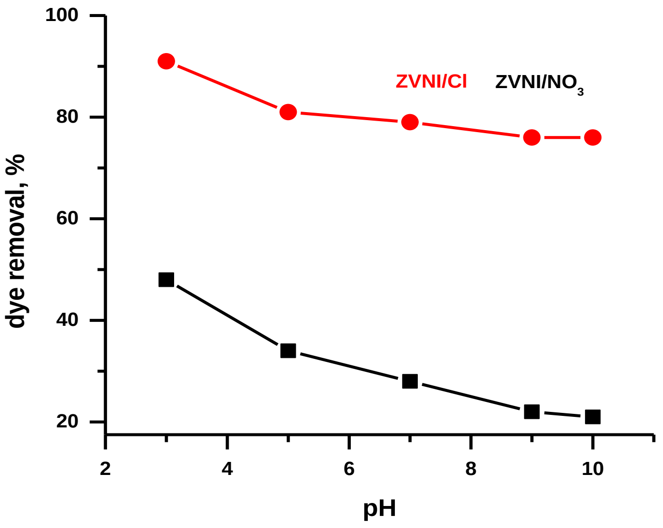

Figure 1. Effect of pH on the adsorption efficiency of ARS dye using ZVNI composites as adsorbents (initial ARS concentration: 600 mg·L^{-1})

Thermodynamic Analysis

To investigate the thermal behavior of the ARS dye adsorption process, experiments were conducted at various temperatures (10°C, 20°C, 33°C, 40°C, and 50°C) using both nZVI/Cl^- and nZVI/NO_3^- composites. The results indicate that adsorption capacity increases as temperature rises, implying an endothermic nature of the process. This observation is supported by the calculated positive enthalpy change (ΔH), suggesting that both adsorption and absorption mechanisms are involved.

An increase in temperature enhances the kinetic energy of ARS dye molecules, promoting their diffusion and enabling deeper penetration into the pores of the adsorbent. Furthermore, the improved interaction between the adsorbent and dye molecules at elevated temperatures suggests a stronger binding affinity, which leads to increased uptake. According to earlier research, traditional thermodynamic equations were used to calculate the thermodynamic parameters, specifically entropy change (ΔS), Gibbs free energy change (ΔG), and enthalpy change (ΔH) [36]. These parameters provide further insight into the feasibility, spontaneity, and energetic profile of the adsorption process.

$$\log\ x_m = \frac{-\Delta H}{2.303\ RT} + \frac{\Delta S}{R} \qquad (3)$$

$$\Delta G = \Delta H - T\Delta S \qquad (4)$$

where Xm is the largest amount of adsorbate (mg/g), R = 8.314 J/mol K (gas constant), and T, measured in Kelvin, is the absolute temperature. As illustrated in Fig. 2, ΔH is identified by the van Hoff plot's slope ($\log(X_m)$ vs 1/T), and ΔS was identified by the intercept.

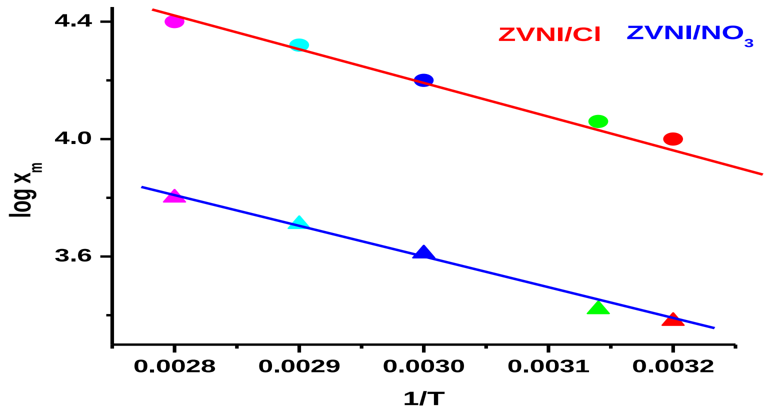

Figure 2 presents the relationship between log Xm and the reciprocal of temperature (1/T) for the adsorption of alizarin red dye onto silica-coated ZVNI/Cl^- and ZVNI/NO_3^- nanocomposites.

From the thermodynamic evaluation, the calculated enthalpy change (ΔH) and entropy change (ΔS) were found to be 24.26 kJ/mol and 69.94 J/(mol·K), respectively. These values suggest that the ARS dye molecules exhibit continued surface mobility, indicating the presence of both adsorption and absorption phenomena. The negative Gibbs free energy change (ΔG = –3.418 kJ/mol at 298 K) confirms that the process is spontaneous and thermodynamically favorable.

Based on enthalpy values, adsorption interactions can generally be classified as follows: (a) physical adsorption for ΔH < 20 kJ/mol, (b) electrostatic interaction when 20 ≤ ΔH ≤ 80 kJ/mol, and (c) chemisorption for ΔH between 80 and 450 kJ/mol [36]. The ΔH values determined in this study fall within the range characteristic of electrostatic interactions, indicating that such forces are primarily responsible for ARS dye adsorption onto both ZVNI composites.

The positive ΔH further confirms that the adsorption is endothermic in nature [37], while the positive ΔS suggests increased randomness at the solid–liquid interface, consistent with the displacement of dye molecules from the aqueous phase onto the solid adsorbent surface [38–40]. **Figure 3** illustrates the experimental adsorption efficiency of ARS dye at different temperatures using NZVI/Cl^- and NZVI/NO_3^- adsorbents. As depicted, higher temperatures result in increased dye removal, which reinforces the endothermic character of the adsorption process.

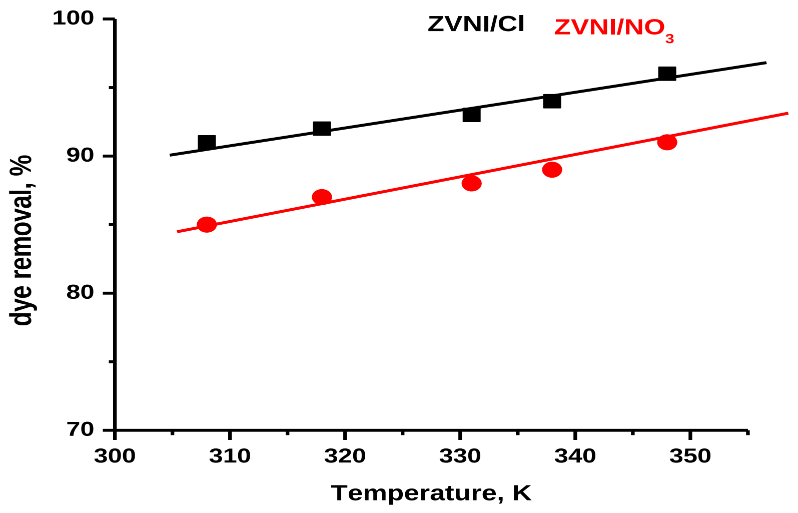

Figure 3 Influence of temperature on the removal efficiency of alizarin red dye using nZVI/Cl^- and nZVI/NO_3^- nanocomposite adsorbents.

Adsorption Isotherms

To better understand the interaction between ARS dye molecules and the surface of the synthesized nanocomposites, adsorption isotherm models were applied to the equilibrium data. Among the widely used models, the **Langmuir** and **Freundlich** isotherms were employed to interpret the experimental results.

Langmuir Isotherm Model

The Langmuir model is based on the premise that adsorption takes place evenly across a surface made up of similar spots, creating a monolayer covering where the adsorbed molecules do not interact [38]. In addition, this model makes the assumption that a dye molecule cannot be adsorbed again at a site after it has taken up residence there. The mathematical form of the Langmuir equation is provided in Equation (5).

$$\frac{C_e}{Q_e} = \frac{1}{a} + \frac{b}{a} C_e \qquad (5)$$

In the Langmuir model, C_e represents the equilibrium concentration of the dye in the liquid phase, while (a) and (b) are the Langmuir constants, which are related to the adsorption energy. *Q*e denotes the equilibrium adsorption capacity of alizarin red dye on the nZVI/Cl^- and nZVI/NO_3^- composites, reflecting the monolayer coverage of the adsorbent surface. Figure 4A shows the relationship between (*C*e/*Q*e) and (*C*e), which was used to fit the adsorption data to the Langmuir model. With monolayer adsorption onto a surface that has similar sites and homogeneous adsorption energy; the results show that the adsorption behaviour is in good agreement with the Langmuir equation [36].

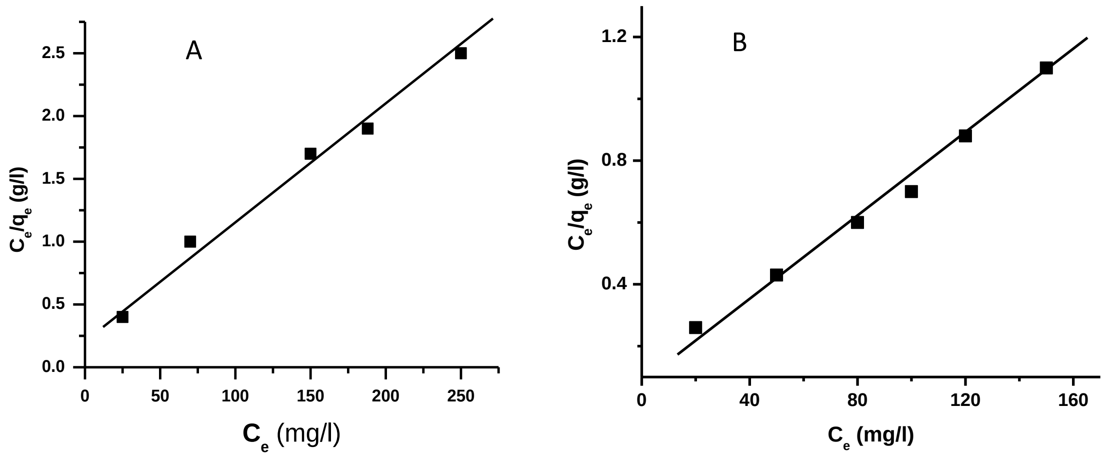

Fig. 4 Langmuir isotherm for the adsorption of alizarin red dye on nZVI/Cl^- (A) and on nZVI/NO_3^- (B)

Freundlich **isotherm model**

The Freundlich equation is given as follows:

$$\boldsymbol{ln\ q_e = ln\ K_f + \frac{1}{n}\ lnC_e} \qquad (6)$$

$$\boldsymbol{K_f = \frac{q_m}{C_o^{1/n}}}$$

In the Freundlich model, K_f and n are constants that represent the adsorption capacity and intensity, respectively. This model is based on the assumption of a heterogeneous surface, where adsorption sites vary in energy, and it does not describe monolayer adsorption. Figure 5A and 5B show the relationship between (ln Q_e)and (ln C_e), which was used to fit the adsorption data of alizarin red dye to the Freundlich equation. The results indicate that the adsorption behavior does not follow the Freundlich model.

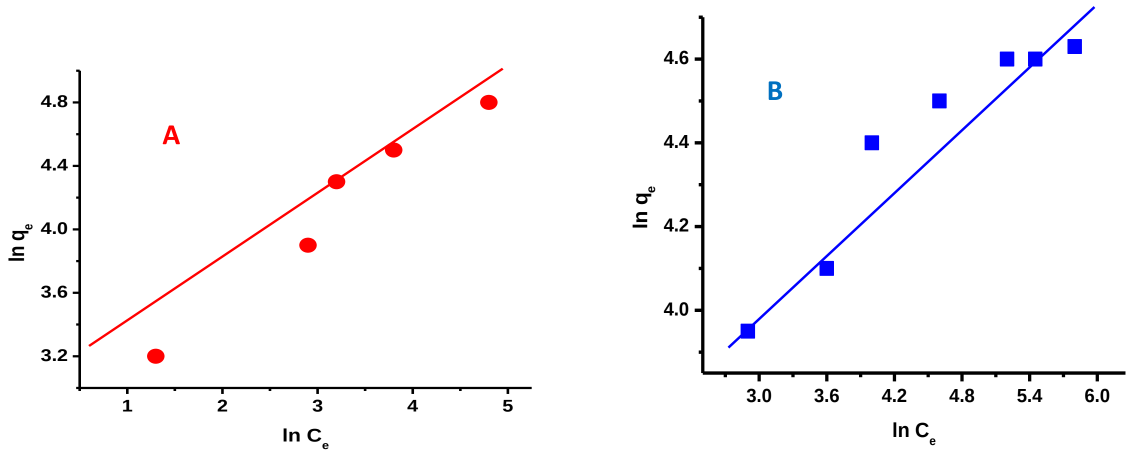

Figure 5 Freundlich isotherm for the adsorption of alizarin red dye onto ZVNI/Cl^- (A) and ZVNI/NO_3^- (B).

Adsorption Kinetics

The adsorption kinetics of alizarin red dye on ZVNI/Cl^- and ZVNI/NO_3^- were analyzed using kinetic models based on experimental data. The rate at which reactant or product concentrations change over time is referred to as kinetics. Both pseudo-first-order and pseudo-second-order

kinetic models were applied to the data. The results indicated that equilibrium for dye adsorption was reached in approximately 80 minutes with 0.1 g of ZVNI/Cl^- and ZVNI/NO_3^- adsorbents [39].

$$\ln(qe - q_t) = \ln qe - k_1\, t \qquad \text{(pseudo-first-order model)}$$

$$\frac{1}{q_t} = \frac{1}{k_2\, q_e} + \frac{t}{q_e} \qquad (\text{pseudo} - \text{second} - \text{order model})$$

where, q_e is the quantity of red alizarin adsorbed in equilibrium (mg / g), and q_t is the quantity of red alizarin adsorbed in various times t (mg / g), respectively, k_1 and k_2 are the kinetic rate constants values. The cinematic data can be correctly characterized with a high correlation coefficient (R2 > 0.9798) in the pseudo-secondary order pattern (Fig. 6)

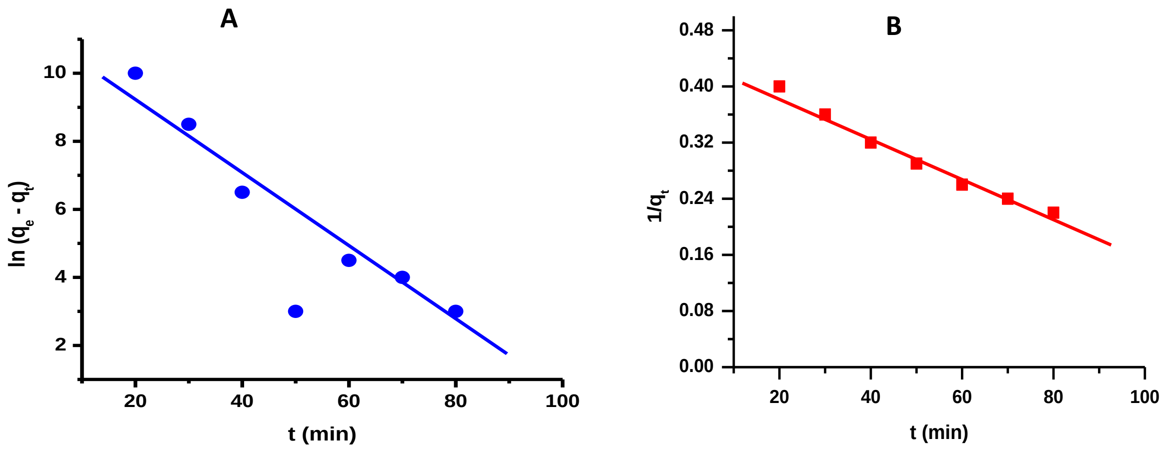

Fig. 6 Dye adsorption kinetic curves, faux first order (A), pseudo second order (B).

Effect of Adsorbent Dose

The impact of varying adsorbent doses on dye adsorption was investigated, and the results are presented in Figure 7. It was observed that increasing the dose of nZVI/Cl^- and nZVI/NO_3^- adsorbents in the dye solution led to a higher removal percentage. This increase is attributed to the greater availability of adsorption sites for dye molecule binding [40,]. Figure 7 demonstrates that the highest removal efficiency was observed at an adsorbent dosage of 3.0 g, achieving 86.7% and 80% dye removal for Alizarin Red-S using nZVI/Cl^- and nZVI/NO_3^- composites, respectively.

Effect of Adsorbent Dose

The dosage of the adsorbent plays a crucial role in determining the adsorption capacity for a given initial concentration of adsorbate. To investigate this, the effect of adsorbent dose on the removal of alizarin red dye was tested using doses ranging from 0.002 to 0.012 g. The experiments were carried out by adding the adsorbent to 50 mL of alizarin red dye solutions with an initial concentration of 150 mg/L for both nZVI/Cl^- and nZVI/NO_3^-. After the systems reached equilibrium, the solutions were centrifuged, and dye concentrations were analyzed. The results, shown in **Figure 7**, indicate that increasing the adsorbent dose enhances the dye removal efficiency, most likely as a result of the adsorbent surface's increased availability of active sites.It was observed that the dye removal efficiency reached a maximum of 96.786% for NZVI/Cl^- and 87.206% for NZVI/NO_3^-.

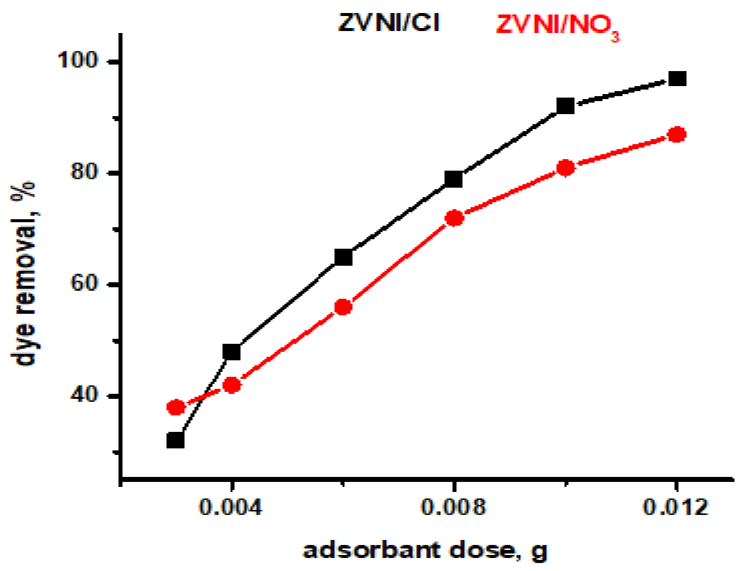

Fig. 7 Effect of adsorbent dose on the alizarin red dye removal efficiency using ZVNI/Cl^- and ZVNI/NO^-_3 adsorbents

Conclusion

The adsorption of Alizarin Red-S dye onto nZVI-based composites is effectively described by the Langmuir isotherm model, indicating monolayer coverage on uniform adsorption sites. Both nZVI/Cl^- and nZVI/NO_3^- demonstrated high removal efficiencies, achieving maximum adsorption capacities of 96.79% and 86.06%, respectively. Thermodynamic parameters

confirmed the spontaneity and endothermic nature of the process, while kinetic analysis revealed that the adsorption follows a pseudo-second-order model. These findings highlight the potential of nZVI composites as efficient and promising materials for the removal of textile dyes from aqueous environments. Given their high performance and environmentally friendly nature, these materials hold strong potential for real-world applications in wastewater treatment, particularly in textile and dyeing industries.

Author contributions I. El-Hallag conceived the original idea and contributed to the conceptualization of the study, data analysis and curation, and manuscript review. A. Al-Owais performed the synthesis of the materials and elucidation of the data and carried out the experimental work. E. El-Mossalamy analysed the data and drafted the manuscript. All authors contributed to the review and editing of the manuscript and approved the final version.

Statements and Declarations

The authors state that none of the work described in this publication could have been influenced by any known competing financial interests or personal relationships.

Funding: Not applicable.

• **Ethical approval:** Not applicable.

• **Informed consent:** Not applicable.

Data availability: All data presented in this study are included in the published article.

Acknowledgements: The authors express their sincere gratitude to the Egyptian Knowledge Bank (EKB) and the Science, Technology & Innovation Authority (STDF) for providing funds for the APC for the publication of this work.

Supplementary Information: The online version contains supplementary material.

Correspondence and requests for materials should be addressed to I. El-Hallag or E. El-Mossalamy.

References

1. Yusuf M, Elfghi F. M, Zaidi S. A, Abdullah E. C, Khan M. A Applications of graphene and its derivatives as an adsorbent for heavy metal and dye removal: a systematic and comprehensive overview,

RSC Advances 5: 50392 (2015). https://doi.org/10.1039/C5RA07223A .
2. Rosi N. L, Giljohann D. A, Thaxton C. S, Lytton-Jean A. K. R, Han M. S, Mirkin C. A Oligonucleotide-Modified Gold Nanoparticles for Intracellular Gene Regulation, Science 312:1027 (2006). doi: 10.1126/science.1125559.
3. El-Naggar F. M, Ali N. M, Moustafa M. M, El-Mossalamy E. H Novel synthesis zero -valent iron nanoparticles for removal alizarin red dye from aqueous solution Benha j. appl. sci. 7: 239-248 (2022). doi: 10. 21608/BJAS.2022.260495.
4. Di L, Chen X, Lu J, Zhou Y, Zhou Y Removal of heavy metals in water using nano zero-valent iron composites: A review Water Process. Eng 53:103913 (2023) https://doi.org/10.1016/j.jwpe.2023.103913
5. Geçgel Ü, Özcan G, Gürpınar G. Ç. Removal of Methylene Blue from Aqueous Solution by Activated Carbon Prepared from Pea Shells (*Pisum sativum*) J Chem 2013: 614083 (2012) https://doi.org/10. 1155/2013/614083.
6. Yang Y, Xu Y, Zhong D, Qiao Q, Zeng H. Efficient removal of Cr(VI) by chitosan cross-linked bentonite loaded nano-zero-valent iron composite: Performance and mechanism J. Hazard. Mater 480: 136183 (2024) https://doi.org/10.1016/j.jhazmat.2024.136183.
7. Taha M. R, Ibrahim A. H. Characterization of nano zero-valent iron (nZVI) and its application in sono-Fenton process to remove COD in palm oil mill effluent J. Environ. Chem. Eng 2: 1-8 (2014) http://dx.doi. org/10.1016/j.jece.2013.11.021.
8. Fan J, Guo Y, Wang J, Fan M Rapid decolorization of azo dye methyl orange in aqueous solution by nanoscale zerovalent iron particles J. Hazard. Mater. 166: 904- 910 (2009). http://dx.doi.org/10.1016/ j.jhazmat.2008.11.091.
9. Rizk R, Hamed M. M. Batch sorption of iron complex dye, naphthol green B, from wastewater on charcoal, kaolinite, and tafla Desalin. Water Treat. 56: 1536 -1546 (2015) https://doi.org/10.1080/ 19443994. 2014.954004.
10. Dada A, lekan A. P, Olatunya A. M Langmuir, Freundlich, Temkin and Dubinin–Radushkevich isotherms studies of equilibrium sorption of Zn^{2+} unto phosphoric acid modified rice Husk IOSR J.Appl. Chem. 3: 38-45 (2012) http://dx.doi.org/10.9790/5736-0313845.
11. Agrawal A, Sahu K. K, Pandey B. D. Removal of zinc from aqueous solutions using sea nodule residue Colloids Surf. A. 237: 133-140 (2004) doi:10.1016/j.colsurfa.2004.01.034.
12. Moha S. V, Karthikeyan J. Removal of lignin and tannin colour from aqueous solution by

adsorption onto activated charcoal Environ. Pollut. 97: 183-187 (1997) doi: 10.1016/s0269-7491(97)00025-0.
13. Mishakov I. V, Bedilo A. F, Richards, R. M, Chesnokov V. V, Volodin A. M, Zaikovskii V. I. Nanocrystalline MgO as a dehydrohalogenation catalyst J.Catal. 206: 40-48 (2002) https://doi.org/10. 1006/ jcat.2001.3474.
14. Richards R. M, Mulukutla R. S, Mishakov I. V, Chesnokov V. V, Volodin A, Zaikovski V, Sun N, Klabunde, K. Nano crystalline ultra-high surface area magnesium oxide as a selective base catalyst Scr. Mater. 44:1663-1666 (2001) https://www.researchgate.net/publication/ 248296923.
15. Wang X. S, Qin Y. Equilibrium Sorption Isotherms for Cu2+ on Rice Bran Process Biochem. 40: 677-680 (2005) https://doi.org/10.1016/j.procbio.2004.01.043.
16. Ghaedi M, Hassanzadeh A, Kokhdan S. N Multiwalled carbon nanotubes as adsorbents for the kinetic and equilibrium study of the removal of alizarin red s and morin. J. Chem. Eng. Data 56: 2511− 2520 (2011) https://doi.org/10.1021/je2000414.
17. Roosta M, Ghaedi M, Mohammadi M. Removal of alizarin Red S by gold nanoparticles loaded on activated carbon combined with ultrasound device: Optimization by experimental design methodology. Powder Technol. 267:134−144 (2014) https://doi.org/10.1016/ j.powtec.2014.06.052.
18. Gholivand M. B, Yamini Y, Dayeni M, Seidi S, Tahmasebi E. Adsorptive removal of alizarin red-S and alizarin yellow GG from aqueous solutions using polypyrrole-coated magnetic nanoparticles. J. Environ. Chem. Eng. 3: 529−540 (2015) https://doi.org/10.1016/j. jece.2015.01.011.
19. Cardoso N. F, Lima E. C, Royer B, Bach M. V, Dotto G. L, Pinto L. A. A, Calvete T. Comparison of Spirulina platensis microalgae and commercial activated carbon as adsorbents for the removal of Reactive Red 120 dye from aqueous effluents. J. Hazard. Mater. 241−242: 146−153 (2012) https://doi.org/10. 1016/ j.jhazmat.2012.09.026.
20. De Lima R. O. A, Bazo A. P, Salvadori D. M. F, Rech C. M, Oliveira D. P, Umbuzeiro G. A Mutagenic and carcinogenic potential of a textile azo dye processing plant effluent that impacts a drinking water source. Mutat. Res. - Genet. Toxicol. Environ. Mutagen 626:53−60 (2007) https://doi.org/ 10.1016/ j.mrgentox.2006.08.002.
21. Bi M, Liang Y, Chi W, Liu H, Chen Y. ChenEnhanced removal of Cr(VI) from aqueous

solutions using a zero-valent nickel/iron-PDA@PVDF membrane prepared via a simple blending method Chem. Eng. J 502: 157891 (2024) https://doi.org/10.1016/j.cej.2024.157891.

22. Jauris I. M, Matos C. F, Saucier,C, .Lima E. C, .Zarbin,A. J. G, Fagan S. B, Machado F. M, .Zanella I. Adsorption of sodium diclofenac on graphene: a combined experimental and theoretical study Phys. Chem. Chem. Phys. 18: 1526−1536 (2016) https://doi.org/10.1039/C5CP05940B.

23. Dos Santos D. C, .Adebayo M. A, Lima E. C, Pereira S. F. P , Cataluña R, Saucier C, Thue P. S, Machado F. M Application of Carbon Composite Adsorbents Prepared from Coffee Waste and Clay for the Removal of Reactive Dyes from Aqueous Solutions. J. Braz. Chem. Soc. 26: 924−938 (2015) https://doi.org/ 10. 5935/ 0103-5053.20150053**.**

24. Saucier C, Adebayo M. A, Lima E. C, Cataluña R, Thue P. S, Prola L. D. T, Puchana-Rosero M. J, Machado F. M, Pavan, F, Dotto G. L. . Microwave-assisted activated carbon from cocoa shell as adsorbent for removal of sodium diclofenac and nimesulide from aqueous effluents. J. Hazard. Mater. 289: 18−27 (2015) http://dx.doi.org/doi:10.1016/ j.jhazmat.2015.02.026.

25. Dotto G. L, Lima E. C, Pinto L. A. A. Biosorption of food dyes onto *Spirulinaplatensis* nanoparticles: Equilibrium isotherm and thermodynamic analysis. Bioresour. Technol. 103: 123−130 (2012) doi: 10.1016/j.biortech.2011.10.038.

26. Dos Santos D. C, Adebayo M. A, Pereira S. F. P, Prola L. D. T, Cataluña R, Lima E. C, Saucier C, Gally C. R, Machado F. M. New carbon composite adsorbents for the removal of textile dyes from aqueous solutions: kinetic, equilibrium, and thermodynamic studies. Korean J. Chem. Eng. 31: 1470−1479 (2014) https://doi.org/10.1007/s11814-014-0086-3.

27. Puchana-Rosero M. J, Adebayo M. A, Lima E. C, Machado F. M, Thue P. S, Vaghetti J. C. P, Umpierres C. S, Gutterres M. Microwave-assisted activated carbon obtained from the sludge of tannery-treatment effluent plant for removal of leather dyes. Colloids Surf. A 504: 105−115 (2016) https://doi.org/ 10.1016/ j. colsurfa.2016.05.059.

28. Machado F. M, Bergmann C. P, Fernandes T. H. M, Lima E. C, Royer B, Calvete T, Fagan S. B. Adsorption of Reactive Red M-2BE dye from water solutions by multi-walled carbon nanotubes and activated carbon J. Hazard. Mater. 192: 1122−1131 (2011) https://doi.org/10.1016/j.jhazmat. 2011.06.020.

29. Gautam R. K, Mudhoo A, Chattopadhyaya M. C. Kinetic, equilibrium, thermodynamic studies and spectroscopic analysis of Alizarin Red S removal by mustard husk. J. Environ. Chem. Eng. 1: 1283−1291 (2013) https://doi.org/10.1016/j.jece.2013.09.021.
30. Namakka M, Rahman M. R, Said K. A. B. M, Muhammad A Insights into micro-and nano-zero valent iron materials: synthesis methods and multifaceted applications RSC Advances 14: 30411-30439 (2024) https://doi.org/10.1039/d4ra03507k.
31. Hasanzadeh M, Simchi A, Shahriyari F. H. Nanoporous composites of activated carbon-metal organic frameworks for organic dye adsorption: Synthesis, adsorption mechanism and kinetics studies J. Ind. Eng. Chem. 81: 405–414 (2020) doi:10.1016/j.jiec.2019.09.031.
32. Niazi A, Ghalie M, Yazdanipour A, Ghasemi J. Spectrophotometric determination of acidity constants of Alizarine Red S in water, water-Brij-35 and water-SDS micellar media solutions. Spectrochim. Acta Part A Mol. Biomol. Spectrosc. 64, 660–664 (2006) https://doi.org/10.1016/j.saa.2005.08.002.
33. ChinY. P, Abdul Raof S. F, Sinniah S, Lee V. S, Mohamad S, Abdul Manan N. S. Inclusion complex of Alizarin Red S with β-cyclodextrin: Synthesis, spectral, electrochemical and computational studies. J. Mol. Struct. 1083: 236–244 (2015) doi:10.1016/j.molstruc.2014.12.010.
34. Saucier C, Adebayo M. A, Lima E. C, Prola L. D. T, Thue P. S, Umpierres C. S, Puchana-Rosero M. J, Machado F. M. Comparison of a homemade Bacury shell activated carbon with MWCNT for the removal of Brilliant Blue FCF food dye from aqueous solutions. Clean: Soil, Air, Water 43: 1389−1400 (2015) https://doi.org/10.1002/clen.201400669.
35. Calvete T, Lima E. C, Cardoso N. F, Dias S. L. P, Ribeiro E. S. Removal of brilliant green dye from aqueous solutions using home made activated carbons. Clean: Soil, Air, Water 38, 521−532 (2010) https://doi. org/101002/clen.201000027.
36. Shi Y, Wang X, Zhong S, Chen W, Feng C, Yang S. Nano zero-valent iron/montmorillonite composite for the removal of Cr(VI) from aqueous solutions: Characterization, performance, and mechanistic insights Applied Clay Science 253: 107345 (2024) https://doi.org/10.1016/j.clay.2024.107345.
37. Rehman R, Mahmud T Sorptive elimination of alizarin red-S dye from water using Citrullus lanatus peels in environmentally benign way along with equilibrium data modeling. Asian J. Chem. 25: 5351− 5356 (2013) doi: 10.14233/ajchem.2013.14179.
38. Fan L, Zhang Y, Li, X, Luo C, Lu F, Qiu H. Removal of alizarin red from water environment

using magnetic chitosan with alizarin red as imprinted molecules. Colloids Surf. B 91: 250−257 (2012) https:// doi.org/10.1016/j.colsurfb.2011.11.014.

39. Fu F, Gao Z, Gao L, Li D. Effective Adsorption of Anionic Dye, Alizarin Red S, from Aqueous Solutions on Activated Clay Modified by Iron Oxide. Ind. Eng. Chem. Res. 50: 9712−9717 (2011) https://doi.org/10.1021/ie200524b.

40. Rizk S, Hamed M. M. Batch sorption of iron complex dye, naphthol green B, from wastewater on charcoal, kaolinite, and tafla Desalin. Water Treat. vol.56: 1536-1546 (2015) https://doi.org/10.1080/ 19443994.2014.954004.
